# Ribonucleotide reductase subunit M2 mediates the mTOR pathway to recruit furin endoprotease and promote maturation of dengue virus

**DOI:** 10.1016/j.isci.2025.113998

**Published:** 2025-11-10

**Authors:** Bouchra Kitab, Michinori Kohara, Kyoko Tsukiyama-Kohara

**Affiliations:** 1 Transboundary Animal Diseases Center, Joint Faculty of Veterinary Medicine, Kagoshima University, Kagoshima-City 890-8580, Japan; 2 Department of Microbiology and Cell Biology, Tokyo Metropolitan Institute of Medical Science, Setagaya-Ku, Tokyo 156-8506, Japan

**Keywords:** Molecular biology, Virology

## Abstract

Dengue virus (DENV) maturation involves calcium-dependent endoprotease furin, which cleaves the precursor membrane (prM) into the M protein in the *trans*-Golgi network, facilitating the release of infectious virus. Here, we demonstrate that ribonucleotide reductase subunit M2 (RRM2) recruits furin to mediate the cleavage of the DENV prM protein. Silencing of *RRM2* reduced furin expression, leading to the accumulation of uncleaved prM in infected cells, increased intracellular DENV RNA, and diminished infectious virus titers in the supernatant. DENV infection prompted colocalization and interaction between RRM2 and furin in hepatoma cells and liver tissues of infected mice, with enhancement of RRM2 expression and furin stability. Silencing *RRM2* impeded the effects of mammalian target of rapamycin (mTOR), leading to decreased furin expression. Additionally, mTOR overexpression reduced uncleaved prM and intracellular viral RNA while increasing furin levels in DENV-infected cells. These findings highlighted a DENV maturation process, which is fine-tuned by the RRM2-mTOR-mediated pathway.

## Highlights

• *RRM2* suppression increases intracellular DENV RNA and reduces viral particle maturation
• RRM2 recruits furin resulting in the cleavage of the viral protein prM
• DENV infection facilitates colocalization and interaction of RRM2 and furin
• mTOR-signaling pathway is involved in the RRM2-mediated furin regulation

## Graphical abstract

**Figure:**
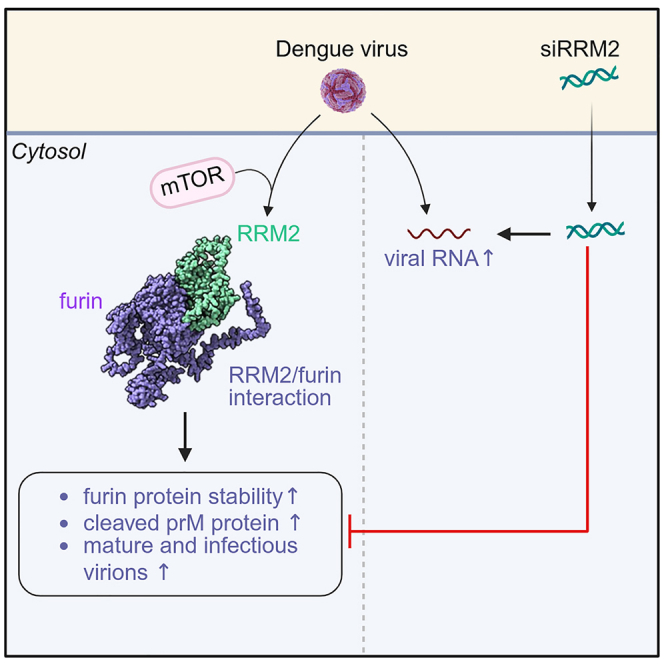

## Teaser

Virology; Molecular biology

## Introduction

With global warming, vector-borne viral diseases, including dengue fever, have increased drastically. Dengue viruses (DENVs), with four distinct serotypes, infect 100–400 million people annually, primarily in tropical and subtropical regions.^1^^,^^2^ DENV is a positive-stranded RNA virus that replicates in the cytoplasm, and immature virions assemble in the endoplasmic reticulum (ER).^3^ These immature non-infectious viral particles contain heterodimers of E and precursor membrane (prM)^4^^,^^5^ and mature via the exocytic pathway in the *trans*-Golgi network (TGN) through the host cell endoprotease furin.^6^^,^^7^ Maturated infectious viral particles are released into the extracellular environment through exocytosis.^8^ We previously found that the hepatitis C virus, belonging to the same *Flaviviridae* family as DENV, utilizes the ribonucleotide reductase subunit M2 (RRM2) to promote its replication by protecting viral NS5B RNA polymerase from hPLIC1-dependent proteasome degradation.^9^

Here, we present a newly clarified role of RRM2 in the DENV life cycle, specifically targeting the viral maturation step. Mammalian ribonucleotide reductase (RR) consists of two large subunits (RR subunit M1, RRM1) and two small RRM2 subunits and catalyzes the production of deoxyribonucleotides (dNTPs) required for DNA synthesis and cell proliferation.^10^ RRM2 is the regulatory component of RR; therefore, research has predominantly focused on investigating RRM2 during infection with DNA viruses and retroviruses. RRM2 modulates the intracellular dNTP pools indispensable for viral DNA synthesis during infection of hepatitis B virus,^11^ human papillomaviruses,^12^ and Kaposi sarcoma-associated herpes virus.^13^ The cyclin-dependent kinase inhibitor p21 inhibits viral cDNA synthesis of HIV-1 and other primate lentiviruses by suppressing RRM2 expression and blocking dNTP biosynthesis.^14^ However, there is still a gap in research exploring RRM2 in the life cycle of RNA viruses, which warrants investigation toward a better understanding of the function of this protein in virus biology, especially those representing a massive disease burden in humans.

## Results

### RRM2 is required for virus maturation and release

**Figure 1:**
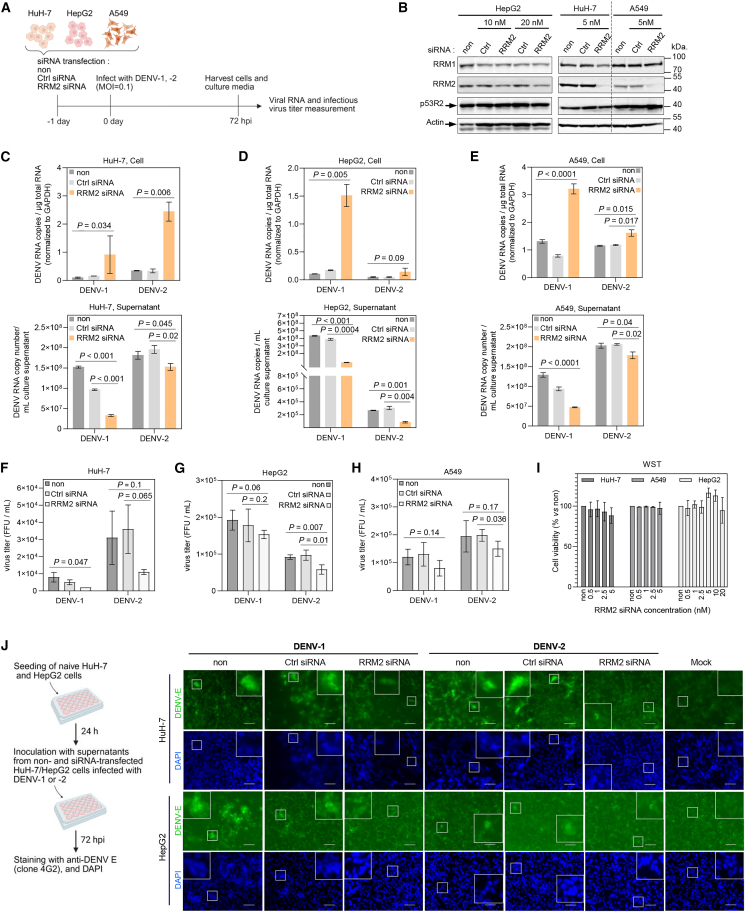
Effect of *RRM2* knockdown on DENV RNA replication and infectious virus production (A) Schematic representation of the experimental protocol. Briefly, HuH-7, HepG2, and A549 cells were transfected with *RRM2* siRNA or non-targeting control siRNA (Ctrl.si) one day before infection with DENV-1 and DENV-2 at a MOI of 0.1. Cells and culture supernatants were harvested 72 h later to measure intracellular and extracellular DENV RNA levels using RT-qPCR and quantify infectious virus titers in culture supernatants via a focus-forming assay. Created using Biorender.com. (B) SDS-PAGE and western blotting were utilized to assess the efficiency of *RRM2* knockdown in HepG2, HuH-7, and A549 cells after treatment with specific *RRM2* siRNA for 96 h at final concentrations of 10, 20, 5, and 5 nM, respectively. The expression levels of other RR subunits, RRM1 and p53R2, were also characterized. β-actin served as a loading control. (C–E) Intracellular and extracellular DENV RNA copy numbers were measured 72 h post-transfection (hpi) via RT-qPCR in HuH-7, HepG2, and A549 cells. Intracellular DENV RNA levels were normalized to *GAPDH* mRNA levels. Results are presented as mean+/-standard deviation (SD). *p* values were assessed using Student’s *t* test. (F–H) Infectious virus titers in culture supernatants from non-transfected and siRNA-transfected HuH-7, HepG2, and A549 cells were determined using a fluorescent focus assay on BHK-21 cells. Results are presented as mean +/- SD. *p* values were evaluated by Student’s *t* test. (I) Viability of HuH-7, HepG2, and A549 cells following treatment with *RRM2* siRNA. Cells were seeded in 96-well plates and transfected with the indicated final concentrations of *RRM2* siRNA. At 96 h post-transfection, cell viability was assessed using the 2(2-methyl-4-nitrophenyl)-3-(4-nitrophenyl)-5-(2, 4disulfophenyl)-2H-tetrazolium, monosodium salt (WST)-8 assay (OD_450_). The percentage of viability was calculated and compared to the untreated control (100% viability). (J) Immunofluorescence microscopy was conducted to evaluate the infection efficiency of DENV particles released from *RRM2* or control siRNA-transfected and non-treated cells. Schematic representation of the experimental procedure. Culture supernatants harvested from non-transfected and siRNA-transfected DENV-infected HuH-7 and HepG2 cells were used to infect naive cell lines at an MOI of 1 (left). Three days post-infection, cell monolayers were analyzed by immunofluorescence for DENV envelope (E) protein expression using anti-flavivirus monoclonal antibody 4G2, followed by Alexa Fluor 488-conjugated goat anti-mouse IgG. Representative immunofluorescence images (400× magnification) depicting DENV-E protein expression in HuH-7 and HepG2 cells. Cell nuclei were stained with DAPI, and cells were observed under a BZ-X700 fluorescence microscope (Keyence Co., Osaka, Japan). White squares indicate enlarged insets. Scale bars, 50 μm (right).

The impact of *RRM2* knockdown on DENV-1 and DENV-2 viral RNA replication was examined using a specific small interfering RNA (siRNA). We used the human hepatoma cell lines HuH-7 and HepG2, as well as the human lung adenocarcinoma cell line A549, all of which are highly susceptible to DENV infection (Figure 1A). The effectiveness of *RRM2* knockdown in each cell line was assessed through western blot analysis. The final concentrations of *RRM2* siRNA were set to 5 nM in both A549 and HuH-7 cells and to 20 nM in HepG2 cells, confirming a significant reduction of RRM2 (Figure 1B). RRM1, which forms an active RR complex with RRM2, along with the RRM2 isoform p53-inducible small subunit p53R2, exhibited no significant changes in protein expression levels, highlighting the specificity of *RRM2* siRNA silencing (Figure 1B). HuH-7, HepG2, and A549 cells were reverse-transfected with *RRM2* siRNA or non-targeting control siRNA one day before infection with DENV-1 and DENV-2 at a multiplicity of infection (MOI) of 0.1. At 72 h post-infection (hpi), both cells and culture supernatants were harvested, and intracellular and extracellular viral RNA copy numbers were quantified by RT-qPCR targeting the DENV non-structural 1 (*NS1*) gene (Figure 1A). For both serotypes, the intracellular viral RNA copy number was substantially higher in *RRM2* siRNA-transfected hepatoma cells compared to those that were non-transfected or transfected with control siRNA (Figures 1C and 1D, upper images). Conversely, *RRM2* knockdown significantly decreased the DENV-1 and DENV-2 viral RNA copy numbers in the culture supernatants compared to those in non-transfected or control siRNA-transfected cells (Figures 1C and 1D, lower images).

A significant elevation in intracellular DENV-1 and DENV-2 viral RNA copy numbers was noted in A549 cells after *RRM2* siRNA treatment (Figure 1E, upper). A decrease in DENV-1 and DENV-2 RNA copy numbers was observed in the culture supernatant (Figure 1E, lower). Consistent with these results, infectious virus amounts were decreased in culture supernatants of *RRM2* siRNA-transfected cells, as determined by the focus-forming assay (Figures 1F–1H). The cytotoxicity of *RRM2* siRNA was evaluated by measuring the viability of cells treated with *RRM2* siRNA for 96 h, demonstrating minimal cytotoxicity at all treatment concentrations (Figure 1I). Therefore, silencing of *RRM2* resulted in a significant increase in intracellular DENV RNA levels while lowering infectious virus titers in the cell culture supernatant across several human cell lines.

We subsequently investigated whether *RRM2* knockdown affected the release of infectious virions into the extracellular environment (Figure 1J). Culture supernatants from HuH-7 and HepG2 cells, non-, control or *RRM2* siRNA-transfected, and subsequently DENV-1- or DENV-2-infected (supernatants from Figures 1C and 1D) were inoculated to naive HuH-7 and HepG2 cells (Figure 1J, left). Three days later, the infected cells were identified through immunofluorescence detection of the DENV envelope (E) protein using the anti-flavivirus monoclonal antibody 4G2 (Figure 1J, right). Cells inoculated with supernatants from *siRRM2*-treated cells showed weak DENV E protein expression, whereas cells inoculated with supernatants from non-transfected or control siRNA-transfected cells expressed abundant DENV E protein (Figure 1J, right). Our observations clearly demonstrated that RRM2 is required for the maturation of DENV or the release of infectious virions into the culture supernatant.

### RRM2 affects the endoprotease furin impacting the cleavage of DENV prM protein

**Figure 2:**
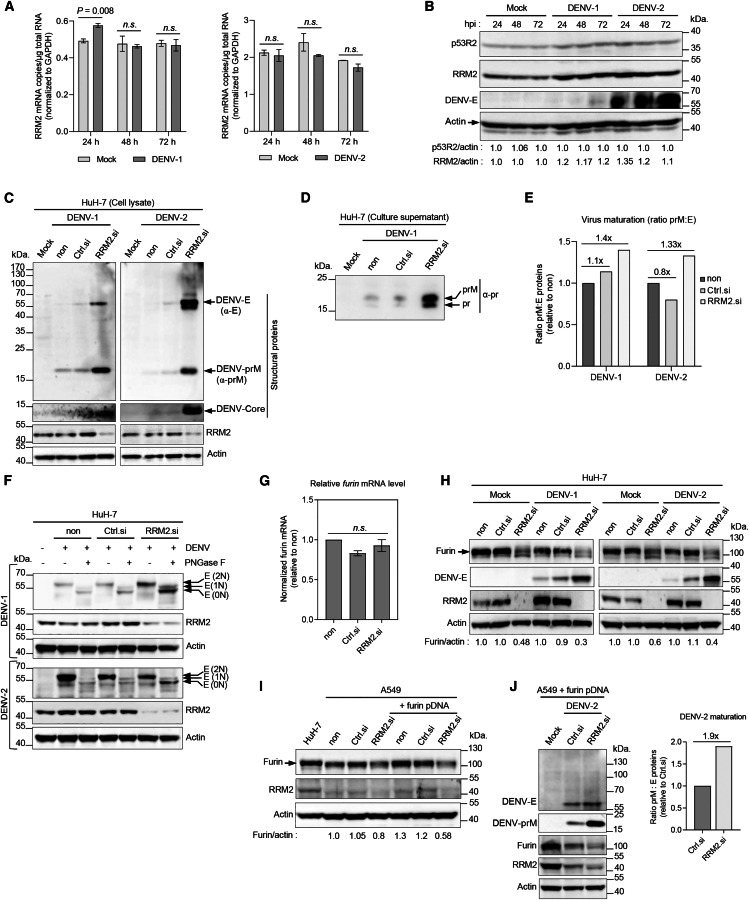
*RRM2* knockdown reduced furin protein levels in the cells, decreasing cleavage of DENV prM protein (A) RT-qPCR analysis of *RRM2* mRNA levels in HuH-7 cells either mock-infected or infected with DENV-1 or DENV-2 at an MOI of 0.1. *RRM2* mRNA levels were normalized to *GAPDH* mRNA levels. Data are expressed as mean ± standard deviation (SD) of triplicate measurements. *p* values were assessed using Student’s *t* test. n.s., not significant. (B) DENV infection increased RRM2 protein levels. HuH-7 cells were mock-infected or infected with DENV-1 or DENV-2 at an MOI of 0.1. Cells were harvested at the indicated time points (24, 48, and 72 hpi) and analyzed by western blotting using anti-RRM2, anti-p53R2, and mouse monoclonal anti-flavivirus E (4G2) antibodies. Endogenous β-actin expression served as an internal control. Protein levels were quantified using ImageJ software and normalized to β-actin levels. Densitometric p53R2/actin and RRM2/actin ratios are shown below the blots. (C) Representative western blot images depict RRM2, DENV envelope (E), DENV capsid, and DENV prM protein expression levels in HuH-7 cells that were transfected with *RRM2* siRNA or control (Ctrl) siRNA (5 nM). HuH-7 cells were either non-transfected or transfected with siRNAs one day prior to infection with DENV-1 or DENV-2 at an MOI of 0.1. At 72 hpi, cells were harvested and subjected to western blot analysis under non-reducing conditions using anti-prM, anti-capsid, anti-flavivirus E (4G2), and anti-RRM2 antibodies. β-actin was utilized as a loading control. (D) Determination of viral maturation was carried out by calculating the ratio of prM to E protein expression, normalized to non-transfected DENV-infected HuH-7 cells. Detected signals of DENV E and prM proteins in (C) were quantified using ImageJ software, and prM-to-E ratios are plotted as bar graphs. Numbers indicate *n*-fold increase compared to non-transfected cells. (E) Western blot analysis of prM and pr proteins in culture supernatants of non-transfected, control siRNA or *RRM2* siRNA-transfected DENV-1 infected HuH-7 cells (C) under reducing conditions using an anti-pr mouse mAb. (F) Analysis of DENV E protein glycosylation status. Mock infected (−) or DENV (+) infected and siRNA-transfected HuH-7 cell lysates were treated with PNGaseF (+) or buffer control (−), then subjected to non-reducing SDS-PAGE and western blotting with anti-E 4G2, anti-RRM2, and anti-actin antibodies. Arrows indicate the positions of undigested and glycosylated E (2N) protein and deglycosylated forms (1N and 0N) of E protein. 0N, 1N, and 2N correspond to the number of N-linked glycans on E protein. β-actin was utilized as a loading control. (G) Quantification of endogenous *furin* mRNA levels relative to *GAPDH* in HuH-7 cells that were non-transfected or transfected with *RRM2* siRNA or control siRNA (Ctrl si) (5 nM) for 96 h. Data are expressed as mean ± SD of triplicate measurements. (H) Effect of *RRM2* knockdown on furin protein expression. HuH-7 cells were either non-transfected or transfected with control siRNA or *RRM2* siRNA one day before mock infection or infection with DENV-1 or DENV-2 at an MOI of 0.1. At 72 hpi, cells were harvested and analyzed by western blotting using anti-furin, anti-RRM2, and anti-DENV-E proteins. β-actin served as a loading control. Protein band intensities were analyzed using ImageJ software. Densitometric furin/actin ratios are shown below the blots. (I) Furin protein levels in HuH-7 and A549 cells with or without transfection of furin expression plasmid (myc-DDK-tagged human furin, 4 μg) and those non-transfected or transfected with control or *RRM2* siRNA (5 nM). Densitometric furin/actin ratios are shown below the blots. (J) A549 cells were seeded in 60 mm dishes, and after 24 h, cells were transfected with the furin expression plasmid (4 μg) alone or co-transfected with *RRM2* or control siRNA. The following day, the cells were infected with DENV-2 at an MOI of 0.1. At 72 hpi, cells were harvested and subjected to western blot analysis under non-reducing conditions using anti-furin, anti-RRM2, anti-prM, anti-flavivirus E (4G2), and anti-actin antibodies. Densitometric prM/actin ratios are shown below the blots (left). Determination of viral maturation was carried out by calculating prM to E protein expression ratios normalized to those of control siRNA-transfected A549 cells. Detected signals of DENV E and prM proteins were quantified using ImageJ software as described in STAR Methods. prM-to-E ratios are plotted as bar graphs. Number indicates *n*-fold increase compared to Ctrl siRNA-transfected cells (right).

We further investigated the detailed role of RRM2 in the DENV maturation process. To evaluate the effect of DENV infection on RRM2 mRNA and protein expression levels, HuH-7 cells were either mock infected or infected with DENV-1 or DENV-2 (MOI = 0.1) and harvested at 24, 48, and 72 hpi (Figure 2A). RT-qPCR showed no significant difference in *RRM2* mRNA levels between mock- and DENV-1- or DENV-2-infected cells (Figure 2A). Western blot analysis indicated that infection with DENV-1 or DENV-2 increased the expression level of RRM2 protein compared to that in mock-infected cells (Figure 2B). The level of DENV E protein accumulated chronologically, indicating viral replication in the cells (Figure 2B). The expression level of p53R2 protein between mock-infected and DENV-infected cells showed no significant difference, suggesting that RRM2 is more functionally important than the p53R2 isoform during DENV infection (Figure 2B).

Next, we characterized the effect of *RRM2* silencing on DENV protein expression (Figure 2C). HuH-7 cells were reverse-transfected with *RRM2* or non-targeting control siRNA before infection with DENV-1 or DENV-2 (MOI = 0.1). At 72 hpi, cell lysates underwent western blot analysis under non-reducing conditions. DENV structural proteins, capsid, prM, and E, accumulated substantially in *RRM2* siRNA-transfected cells compared to non-transfected and control siRNA-transfected cells (Figure 2C). This is consistent with the impairment of DENV maturation and viral RNA accumulation in cells via *RRM2* knockdown, as shown in Figure 1. DENV E protein was detected exclusively in its monomeric form (∼60 kDa) under non-reducing conditions (Figure 2C). Immunoblot analysis of the DENV “pr” fragment in the culture supernatants of non- and siRNA-transfected DENV-1-infected-HuH-7 cells revealed that less amount for pr (∼17 kDa) than for prM (∼21 kDa) was detected in the culture supernatant of *RRM2* siRNA-transfected cells (Figure 2D), suggesting reduced levels of prM cleavage, and therefore, an increased amount of immature prM-containing virions is present. The high amount of DENV prM protein in *RRM2* siRNA-transfected cells indicates that prM protein remains markedly more uncleaved in *RRM2* siRNA-transfected cells than in control cells. During the flavivirus life cycle, prM proteins on assembled virus particles are cleaved by the host protease furin into “pr” peptide and a virion-associated “M” protein during export through the secretory pathway to reach the cell surface, leading to virus maturation.^6^^,^^7^^,^^8^ Immunoblotting analysis (Figure 2C) was used to characterize the maturation status of intracellular DENV particles by calculating the ratio of the intensity of the prM protein band to that of the E protein band (Figure 2E). More DENV-1 and DENV-2 particles were produced in *RRM2* siRNA-transfected HuH-7 cells with prM:E ratios 1.4-fold and 1.33-fold higher than those in non-transfected cells, respectively, indicating suppression of viral maturation (Figure 2E). Taken together, these results suggest that *RRM2* knockdown suppresses the cleavage of DENV prM, and consequently, more immature prM-containing viral particles accumulate in infected cells. Interestingly, the failure to cleave prM in flaviviral particles leads to the release of non-infectious virions.^15^^,^^16^ Thus, the decreased level of infectious viral particles released from DENV-infected cells treated with *RRM2* siRNA was likely due to the reduced prM cleavage.

DENV E protein contains two N-linked glycosylation sites (N67 and N153), which are present in most strains of the four DENV serotypes.^6^^,^^7^^,^^8^ To verify the glycosylation status of DENV E, siRNA-transfected and DENV-infected HuH-7 cell lysates were untreated or treated with the peptide N-glycosidase F (PNGase F) to remove N-linked glycans, then analyzed by western blotting using the anti-E 4G2 mAb under non-reducing conditions (Figure 2F). DENV-E proteins produced in *RRM2* siRNA-treated cells displayed differing digestion patterns compared with non- and control siRNA-treated cells. A high amount of digested E protein from *RRM2* siRNA-treated cells displayed lower molecular weights after PNGase F treatment, indicating glycan cleavage (Figure 2F). This result indicates an enhancement of DENV E protein glycosylation state by *RRM2* siRNA treatment. Next, the subcellular localization of DENV-2 E glycoprotein by immunofluorescence assay in *RRM2* siRNA-treated HuH-7 cells by co-labeling with cellular markers calnexin (ER), ERGIC-53 (ER-Golgi intermediate compartment), GM130 (*cis*-Golgi), and TGN46 (*trans-*Golgi network) (Figure S1). The E protein was highly enriched in the ER both in control and *RRM2* siRNA-treated cells. Notably, enhanced localization of E protein in the Golgi apparatus and TGN was observed in *RRM2* siRNA-treated cells compared to the control cells, suggesting that it has been induced by increased retention of prM and E viral proteins (Figure S1).

To understand the mechanism linking RRM2 expression to the reduced cleavage efficiency of DENV prM, we hypothesized that RRM2 targets furin expression or processing activity. Furin is a type I transmembrane protein belonging to the subtilisin/kexin-like proprotein convertase family that catalyzes the proteolytic maturation of many precursor proteins within the secretory pathway.^17^ Furin cycles between the plasma membrane, endosomes, and TGN, maintaining its predominant localization in TGN.^18^ First, we examined the effect of *RRM2* knockdown on *furin* transcription using RT-qPCR. *Furin* mRNA expression levels in HuH-7 cells were measured 72 h after *RRM2* siRNA transfection and no substantial difference was observed compared to control siRNA-transfected cells (Figure 2G). Importantly, western blot analysis using the anti-furin antibody (PA1-062) revealed a marked decrease in furin protein levels in *RRM2* siRNA-transfected HuH-7 cells that were either mock-, DENV-1-, or DENV-2-infected (Figure 2H). We validated these results using A549 cells (Figure 2I). Notably, Tay et al.^19^ reported that A549 cells expressed lower levels of furin than HuH-7 cells, which is consistent with our results (Figure 2I). *RRM2* knockdown decreased furin protein levels in A549 cells, which was less pronounced than that in HuH-7 cells (Figure 2I). Therefore, we co-transfected the siRNAs in combination with a furin expression plasmid into these cells. Compared to control cells, *RRM2* knockdown resulted in a striking decrease in furin protein levels in furin-overexpressing cells (Figures 2I and 2J), along with a concomitant increase in intracellular DENV prM levels (Figure 2J, left) and an increased prM:E ratio, indicating impaired viral maturation (Figure 2J, right).

### DENV infection facilitates RRM2-furin interaction

**Figure 3:**
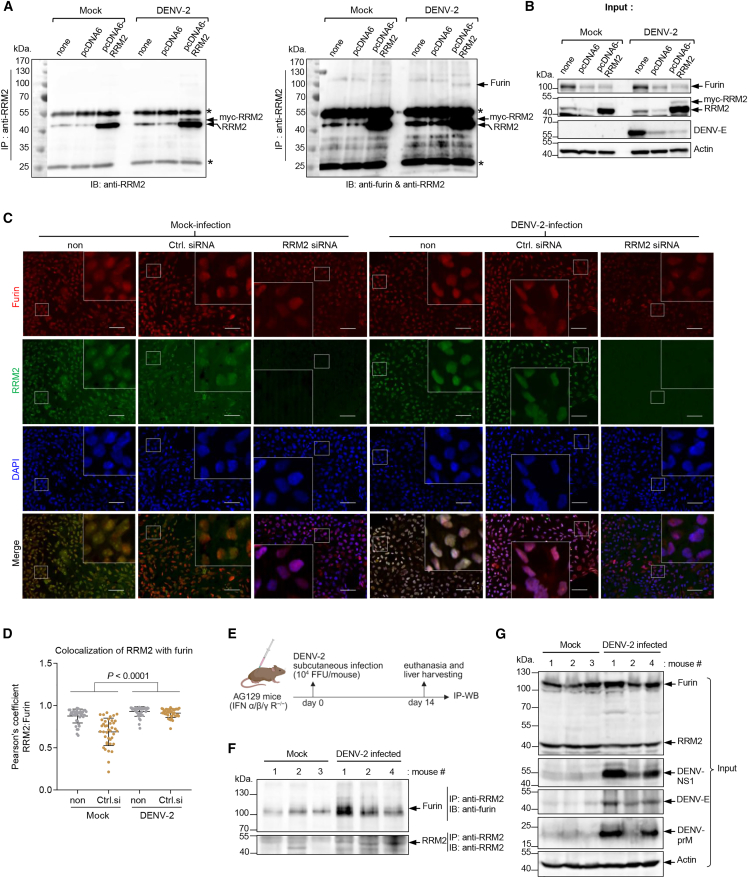
RRM2 colocalizes and interacts with furin in DENV-infected cells (A) Analysis of RRM2 and furin interactions through immunoprecipitation. HuH-7 cells were either mock-transfected (non) or transfected with the expression plasmid for RRM2 (pcDNA6/myc-His-RRM2) or the empty vector pcDNA6/myc-His (5 μg) using Lipofectamine LTX (Invitrogen), followed by either mock-infection or infection with DENV-2 (MOI = 0.1). At 48 hpi, cell lysates were precipitated with anti-RRM2 antibody, and the expression levels of RRM2 and furin in the precipitates were analyzed via immunoblotting using mouse anti-RRM2 (left and right) and rabbit anti-furin (right) antibodies. ∗IgG heavy and light chains. (B) Western blot analysis of the inputs from each cell lysate. β-actin served as a loading control. (C) Representative confocal microscopy images showing the subcellular localization and expression of RRM2 and furin in *RRM2* siRNA-treated cells compared to those in untreated (non) and control siRNA-treated cells. HuH-7 cells were either mock-infected or infected with DENV-2 following transfection with *RRM2* or control siRNA. On day 3 post-infection, cells were stained with anti-furin and anti-RRM2 antibodies and detected using Alexa Fluor 488- and Alexa Fluor 568-conjugated secondary antibodies, respectively. Cell nuclei were stained with DAPI. Cells were examined using a fluorescence microscope, BZ-X700 (Keyence Co., Osaka, Japan). Images were captured at 200× magnification. Scale bars, 50 μm. White squares denote enlarged insets. The z stack was set to 0.3 μm. (D) Quantification of colocalization between RRM2 and furin in mock-infected and DENV-2-infected cells was based on Pearson’s correlation coefficient using BIOP JACoP (Fiji/ImageJ software) (*n* = 40 cells per group). Black lines represent mean ± SD. Statistical analysis was performed using two-way ANOVA. (E) Schematic representation of the experimental design. Female AG129 mice (5 weeks old) were subcutaneously infected with DENV-2 (10^4^ focus-forming units/mouse), and at 14 days post-infection, mice were euthanized, and liver tissues were collected for immunoprecipitation and western blot analysis. Mock-infected AG129 mice were used as controls. (F) Analysis of RRM2 and furin interactions in mouse liver tissues through immunoprecipitation. Tissue lysates were precipitated with anti-RRM2 antibody, and the hepatic protein expression levels of RRM2 and furin in the precipitates were analyzed via immunoblotting using mouse anti-RRM2 and rabbit anti-furin antibodies. (G) Hepatic protein expression levels of RRM2, furin, DENV-NS1, -E, and -prM in the inputs of liver tissue lysates were determined via western blot analysis. β-actin served as a loading control.

To clarify the mechanism by which *RRM2* silencing modulates furin protein levels, we examined the interaction between RRM2 and furin using immunoprecipitation (Figures 3A and 3B). HuH-7 cells were transfected with the RRM2 expression plasmid (pcDNA6/myc-His-RRM2) or the empty vector control pcDNA6/myc-His 24 h prior to either mock infection or infection with DENV-2 (MOI = 0.1). Cells were harvested 48 h after infection, and cell lysates were immunoprecipitated using an anti-RRM2 antibody. The precipitated material was analyzed by immunoblotting with anti-RRM2 (Figure 3A, left), anti-RRM2, and anti-furin antibodies (Figure 3A, right). An approximately 98-kDa band corresponding to furin was observed in DENV-infected cells and immunoprecipitated with RRM2 (Figure 3A, right). In mock-infected cells, anti-RRM2 antibodies yielded myc-tagged RRM2 precipitates, but furin protein was not detected (Figure 3A, right). These observations indicate that RRM2 interacts with furin during DENV infection. This result was further verified using an immunofluorescence assay (Figure 3C). A change in the subcellular location of RRM2 was noted, where it colocalized with furin in DENV-2 infected cells (Figure 3C), yielding a Pearson’s correlation coefficient of 0.91 compared to 0.78 in mock-infected cells (*p* < 0.0001, two-way ANOVA) (Figure 3D). Furin was retained in the TGN in both mock-infected and DENV-2-infected cells (Figure S2A), whereas an increased localization of RRM2 in the TGN was observed in DENV-2 infected cells (Figure S2B). These observations are also consistent with the western blot results showing that RRM2 protein levels increased in DENV-infected cells (Figures 2B and 3B). We further investigated whether RRM2 interacts with furin *in vivo* during DENV infection. Therefore, types I and II interferon receptor knockout mice, AG129, were mock-infected or subcutaneously infected with DENV-2, and after 14 days post-infection, liver tissues were harvested for immunoprecipitation (Figure 3E). Liver tissue lysates were incubated with anti-RRM2 antibodies, and the resulting immunoprecipitates were analyzed by western blotting (Figure 3F). Furin and RRM2 were co-precipitated in mouse liver lysates and more precipitated in the presence of DENV infection (Figures 3F and 3G). To gain more insight into the RRM2/furin protein complex, a modeling of their interaction structure was obtained using AlphaFold3-multimer (Figures S3A and S3B), which supported their possible interaction. Collectively, these results highlight the functional significance of RRM2 during DENV maturation through its interaction with furin *in vitro* and *in vivo*.

### RRM2 expression is important for furin protein stability

**Figure 4:**
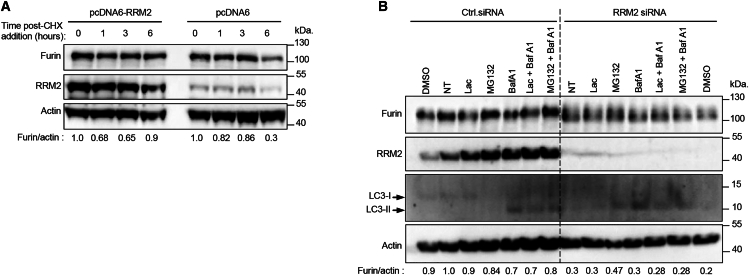
RRM2 expression is important for furin protein stability (A) RRM2 overexpression enhances furin protein stability. HuH-7 cells were plated in 60 mm dishes and transfected with the RRM2-encoding plasmid (pcDNA6/myc-His-RRM2, 5 μg) or the empty vector pcDNA6/myc-His (5 μg) as a negative control. At 72 h post-transfection, cells were treated with the protein synthesis inhibitor CHX (100 μg/mL) for 1, 3, and 6 h. Cells were harvested and analyzed by western blotting using anti-furin, anti-RRM2, and anti-actin antibodies. Protein band intensities were evaluated using ImageJ software. Densitometric furin/actin ratios are displayed below the blots. (B) Effects of specific proteasome and lysosome inhibitors on furin stability. HuH-7 cells were transfected with *RRM2* or control siRNA for 72 h and then either left untreated (NT) or treated with the proteasome inhibitors lactacystin (Lac, 5 μM), MG-132 (10 μM), the lysosome inhibitor bafilomycin A1 (Baf A1, 20 nM), DMSO, or combinations of these inhibitors for 6 h prior to harvest. Cell lysates underwent western blot analysis using anti-furin and anti-RRM2 antibodies. β-actin was utilized as a loading control. An anti-LC3I/II antibody was employed to assess lysosomal inhibition by bafilomycin A1. An increase in LC3-II protein levels indicates the effect of bafilomycin A1. Densitometric furin/actin ratios are presented below the blots.

In order to determine whether RRM2 regulates furin expression via translational or post-translational modifications, we analyzed furin stability using a cycloheximide chase assay. HuH-7 cells were transfected with the RRM2 expression plasmid pcDNA6-myc-His-RRM2 or the empty vector pcDNA6 as control, and at 72 h post-transfection, cells were treated with the protein synthesis inhibitor cycloheximide (CHX, 100 μg/mL) at the indicated times (Figure 4A). RRM2 overexpression promoted furin stability after treatment with cycloheximide for 6 h, whereas furin degradation was enhanced in pcDNA6-transfected cells (Figure 4A). These results indicate that RRM2 expression is required to maintain furin protein stability. There are two main pathways involved in intracellular protein degradation in eukaryotic cells: the ubiquitin-proteasome system and the autophagy-lysosome pathway. To examine whether the resulting decrease in furin protein levels upon *RRM2* silencing was due to proteasomal- or lysosomal-mediated proteolysis, HuH-7 cells were transfected with control or *RRM2* siRNA for 72 h and then treated with the proteasome inhibitors lactacystin and MG132 and the lysosome inhibitor bafilomycin A1 or both for the last 6 h prior to harvest (Figure 4B). Western blot analysis revealed that *RRM2* siRNA treatment markedly reduced furin protein levels in HuH-7 cells, whereas treatment with proteasome or lysosomal inhibitors did not rescue furin levels (Figure 4B). These results suggest that the decreased furin protein levels upon *RRM2* silencing were not due to proteasome- or lysosome-mediated proteolysis, indicating the existence of other regulatory pathways.

### RRM2-mTOR pathway regulates DENV maturation process

**Figure 5:**
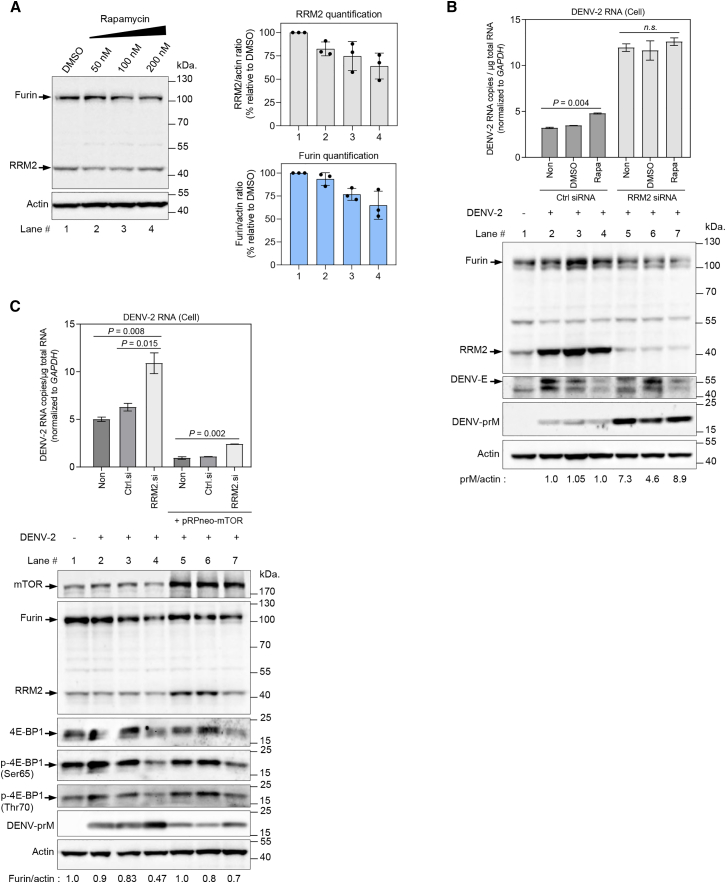
RRM2 regulates furin protein, DENV-prM, and intracellular viral RNA levels through mTOR-dependent pathway (A) Immunoblot analysis of furin and RRM2 protein expression in HuH-7 cells after treatment with the mTOR inhibitor rapamycin. Cells were seeded in a 12-well culture plate and treated after 24 h with rapamycin (Rapa) at escalating concentrations (50, 100, and 200 nM) or DMSO for an additional 12 h. Cells were harvested and analyzed by western blotting with anti-furin, anti-RRM2, and anti-actin antibodies (left). Densitometric analysis of RRM2 (upper) and furin (lower) relative to β-actin is expressed as a percentage of DMSO treatment (lane 1) using ImageJ software and displayed as bar graphs (right). Quantification data are expressed as mean ± standard deviation (SD) from three independent experiments. (B) The combination of *RRM2* siRNA transfection and rapamycin treatment synergistically reduced intracellular furin protein levels. HuH-7 cells were transfected with *RRM2* siRNA or control siRNA one day prior to infection with DENV-2 at an MOI of 0.1. At 48 hpi, cells were treated with DMSO or rapamycin (200 nM) for an additional 12 h. Cells were harvested to isolate intracellular viral RNA and total protein for RT-qPCR and western blot analysis, respectively. Intracellular DENV RNA levels were assessed via RT-qPCR (DENV *NS1*) and normalized to those of *GAPDH*. Significant *p* values were determined using Student’s *t* test (upper). Cell lysates were analyzed via western blotting using anti-furin, anti-RRM2, anti-E (4G2), anti-prM, and anti-actin antibodies. prM protein levels were quantified using ImageJ software and normalized to β-actin levels. Densitometric ratios of prM/actin are depicted below the blots (lower). (C) Effect of mTOR overexpression on furin expression. HuH-7 cells were transfected with *RRM2* siRNA or control siRNA with or without the mTOR expression plasmid (pRP[Exp]-Neo-CAG>hMTOR, 2.5 μg), followed by infection with DENV-2 at an MOI of 0.1. At 48 hpi, cells were harvested to isolate intracellular viral RNA and total protein for RT-qPCR and western blot analysis, respectively. Intracellular DENV RNA levels were measured by RT-qPCR (DENV *NS1*) in HuH-7 cells and normalized to *GAPDH* mRNA levels. Significant *p* values were calculated using Student’s *t* test (upper). Expression levels of mTOR, furin, RRM2, 4E-BP1, phospho-4E-BP1 (Ser65), phospho-4E-BP1 (Thr70), and prM were assessed by immunoblotting of total cell lysates with anti-mTOR, anti-RRM2, anti-furin, anti-4E-BP1, anti-phospho-4E-BP1 (Ser65), anti-phospho-4E-BP1 (Thr70), and anti-prM antibodies. β-actin was employed as a loading control. Densitometric ratios of furin/actin are indicated below the blots (lower).

The mammalian target of the rapamycin (mTOR) pathway regulates several signaling networks involved in maintaining cell growth and homeostasis.^20^ The supply of nutrients and growth factors to cells increases mTOR activity, which promotes protein synthesis and suppresses overall proteolysis.^21^ In pancreatic cancer, silencing *RRM2* inactivates the PI3K/AKT/mTOR pathway, and reciprocal regulation of RRM2 and mTOR occurs in mammalian cells.^22^ P53 suppresses RRM2 by inhibiting mTORC1 in cancer cell lines, and eukaryotic translation initiation factor 4E-binding proteins (4E-BP) 1 and 2 double knockout mice demonstrated elevated RRM2 levels.^23^ To evaluate the effects of mTOR inhibition on furin protein expression, HuH-7 cells were treated with increasing concentrations of rapamycin (50, 100, and 200 nM) or Dimethyl sulfoxide (DMSO) as a control (Figure 5A). Cells were harvested for western blot analysis 12 h after treatment. Rapamycin treatment resulted in a dose-dependent decrease in furin protein levels, with a concomitant decrease in RRM2 protein levels (Figure 5A). In addition, combining *RRM2* siRNA transfection with rapamycin treatment resulted in a synergistic effect in decreasing furin protein levels and increasing intracellular DENV-2 prM protein levels compared to those under *RRM2* siRNA treatment without rapamycin (Figure 5B, lower, lane 5 or 6 versus lane 7). Rapamycin treatment increased intracellular DENV-2 viral RNA levels in the control siRNA treatment (Figure 5B, upper, left). In *RRM2* siRNA-transfected HuH-7 cells (Figure 5B, upper, right), intracellular DENV-2 viral RNA levels increased, and no significant additive effect of rapamycin treatment was observed. This suggests that *RRM2* knockdown and rapamycin may act through overlapping pathways to increase intracellular DENV viral RNA levels and impair viral maturation. After confirming the effect of mTOR inhibition on furin expression, we examined furin expression upon activation of the mTOR pathway. HuH-7 cells were transfected with the mTOR expression plasmid, either alone or co-transfected with *RRM2* or control siRNA, followed by infection with DENV-2 at an MOI of 0.1. At 48 hpi, the cells were harvested to isolate intracellular viral RNA and total protein for RT-qPCR and western blot analysis, respectively. As shown in Figure 5C, RRM2 protein levels were upregulated in mTOR-overexpressing cells, consistent with previous reports.^22^^,^^23^ Importantly, mTOR overexpression rescued furin protein levels in *RRM2* siRNA-transfected cells, together with a decrease in 4E-BP1 expression levels (Figure 5C, lower, lane 4 versus lane 7). These observations are in line with a previous study showing that functional inhibition or knockdown of RRM2 directly affects protein translation through the loss of the inhibitory phosphorylation of 4E-BP1^24^ and indicate that the mTOR-4E-BP1 signaling pathway should be involved in stabilizing furin protein levels in *RRM2* siRNA-transfected cells. This stabilization was accompanied by decreased intracellular DENV RNA levels (Figure 5C, upper) and a reduction in DENV prM protein levels (Figure 5C, lower, lane 4 versus lane 7), suggesting enhanced DENV prM cleavage by furin.

## Discussion

**Figure 6:**
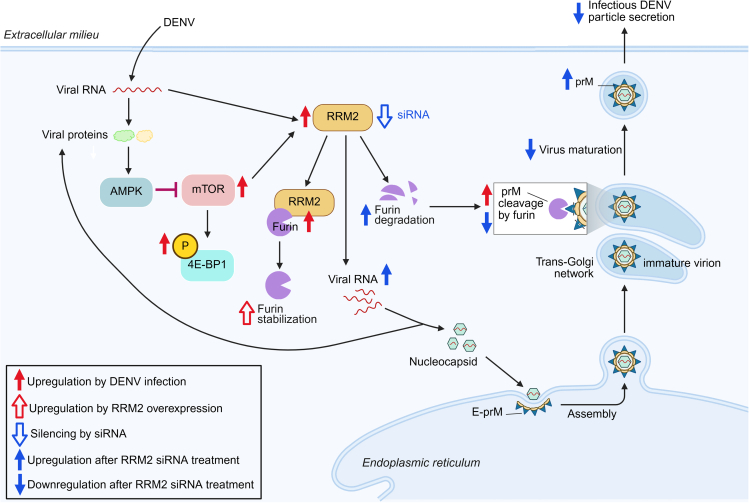
Schematic illustration of the mechanism underlying RRM2 regulation of furin protein levels and DENV maturation Treatment with *RRM2* siRNA increased intracellular DENV RNA levels and promoted furin protein degradation through an mTOR/4E-BP1-dependent pathway, leading to elevated levels of uncleaved prM protein in DENV particles and hindering viral maturation. In contrast, DENV infection induced colocalization and interaction between RRM2 and furin in cells, and RRM2 overexpression, which enhanced furin stability. Created using Biorender.com.

The interaction of DENV with mTOR signaling is essential during DENV replication and induction of antiviral immune responses. However, this interaction has not been thoroughly analyzed, and there are conflicting results regarding mTOR activation in DENV-infected cells.^25^^,^^26^^,^^27^ DENV transiently activates the metabolic regulator 5′-adenosine-monophosphate-activated kinase (AMPK) while inhibiting mTOR to promote viral replication.^26^ DENV NS1 protein interacts with AMPK to activate the AMPK/ERK/mTOR pathway and induce autophagy.^27^ Taken together, our results are consistent with a model wherein siRNA-mediated knockdown of *RRM2* promotes proteolysis of the endoprotease furin, resulting in decreased furin intracellular levels, which reduces the cleavage of the DENV prM protein and consequently the maturation of progeny virions (Figure 6). In contrast, DENV infection regulates the mTOR/4E-BP1 pathway and induces the interaction of RRM2 with furin and RRM2 overexpression, which promotes furin stability and cleavage of prM protein (Figure 6).

In addition to the DENV prM protein, the role of RRM2 in regulating furin expression can be extended to other flaviviruses. Furin cleaves many mammalian proprotein substrates at multibasic cleavage sites to yield functional proteins, cell surface receptors, adhesion molecules, cytokines, hormones, and growth factors.^28^ Therefore, dysregulation of furin expression or activity has been linked to various pathological conditions, including Alzheimer’s disease and cancer.^28^ This reflects the importance of RRM2 in pathophysiological settings involving furin. Furin is also involved in the proteolytic activation of bacterial exotoxins and viral glycoproteins.^28^ Cleavage of viral glycoprotein precursors by furin is a crucial step in the replication and maturation of many viruses from diverse families, such as gp160 of HIV-1,^29^ hemagglutinin in avian influenza virus,^30^ secreted e antigen of hepatitis B virus,^31^ and the spike protein of the coronavirus SARS-CoV-2.^32^ The acquisition of a furin cleavage site is the primary determinant of viral virulence and pathogenicity.^33^^,^^34^ For example, the furin cleavage site is a distinguishing feature of SARS-CoV-2 compared to other SARS-related coronaviruses, and this site is involved in SARS-CoV-2 host range expansion.^32^

Based on these data, viruses that depend on furin processing activity may also utilize RRM2 during the viral life cycle. The identification of cellular factors that contribute to the late steps of the viral replication cycle, such as virus maturation, is highly valuable since these factors could impact viruses from the same or different families and may represent potential broad-spectrum antiviral targets. In conclusion, to the best of our knowledge, the present study demonstrates the involvement of RRM2 in regulating intracellular furin protein stability and expression, which is essential for proper cleavage of the DENV prM protein during viral maturation. Thus, this study highlights that RRM2 is a vital host factor for viral maturation and release.

### Limitations of the study

In future studies, it would be important to validate the effect of RRM2 on furin expression and prM cleavage during infection with other flaviviruses.

## Resource availability

### Lead contact

Further information and requests for resources should be directed to and will be fulfilled by the lead contact, Dr. Kyoko Tsukiyama-Kohara (kkohara@vet.kagoshima-u.ac.jp).

### Materials availability

All materials generated in this study are available from the lead contact.

### Data and code availability

• All data reported in this paper will be shared by the lead contact upon request.
• This paper does not report original code.

## Acknowledgments

We thank Prof. Kouichi Morita and Dr. Masayuki Saijou for providing the DENV-1 and DENV-2 strains and Editage (www.editage.jp) for English edition. This study was supported by the grants from 10.13039/100009619Japan Agency for Medical Research and Development (Grant No. JP253fa827017); 10.13039/100019702Tokyo Metropolitan Government; 10.13039/501100003478Ministry of Health, Labour and Welfare; and 10.13039/501100001700Ministry of Education, Culture, Sports, Science and Technology Japan.

## Author contributions

Conceptualization, B.K. and K.T.-K.; methodology, B.K., M.K., and K.T.-K.; data curation, B.K.; data analysis and interpretation, B.K., M.K., and K.T.-K.; visualization, B.K., M.K., and K.T.-K.; validation, B.K., M.K., and K.T.-K.; writing – original draft, B.K.; writing – review and editing, B.K., M.K., and K.T.-K.; supervision, M.K. and K.T.-K.; funding acquisition, M.K. and K.T.-K.

## Declaration of interests

The authors declare no competing interests.

## STAR★Methods

### Key resources table

**Table:** 

REAGENT or RESOURCE	SOURCE	IDENTIFIER
**Antibodies**		

Rabbit polyclonal anti-furin	Thermo Fisher Scientific	Cat# PA1-062; RRID:AB_2105077
Mouse monoclonal anti-RRM2 (clone 1E1)	Sigma-Aldrich	Cat# WH0006241M1; RRID:AB_1843447
Rabbit polyclonal anti-RRM2	GeneTex	Cat# GTX103193; RRID:AB_2037925
Rabbit polyclonal anti-RRM1	Abcam	Cat# ab137114
Goat polyclonal anti-p53R2 (clone *N*-16)	Santa Cruz Biotechnology	Cat# sc-10840; RRID:AB_653758
Mouse anti-flavivirus envelope protein monoclonal antibody (D1-4G2-4-15)	ATCC	CVCL_J890
Rabbit polyclonal anti-DENV capsid	GeneTex	Cat# GTX103343; RRID:AB_1240697
Rabbit polyclonal anti-DENV prM	GeneTex	Cat# GTX128093; RRID:AB_2885700
Mouse monoclonal anti-DENV pan-serotype prM (clone CC5)	The Native Antigen Company	Cat# MAB12136
Mouse monoclonal anti-DENV pr (clone 47-2)	LEADGENE	Cat# LDG0013YA
Mouse monoclonal anti-DENV NS1 (clone DN1)	Abcam	Cat# ab41490; RRID:AB_869446
Rabbit polyclonal anti-calnexin	Stressgen	Cat# SPA-860
Rabbit monoclonal anti-GM130	Abcam	Cat# ab52649; RRID:AB_880266
Sheep polyclonal anti-Human TGN46	Bio-Rad	Cat# AHB500GT; RRID:AB_2203291
Rabbit polyclonal anti-ERGIC-53/p58	Sigma-Aldrich	Cat# E1031; RRID:AB_532237
Rabbit polyclonal anti-mTOR	GeneTex	Cat# GTX101557; RRID:AB_1241080
Rabbit polyclonal anti-LC3I/II	MBL International	Cat# PM036; AB_2274121
Rabbit polyclonal anti-4E-BP1	Cell Signaling Technology	Cat# 9452; RRID:AB_331692
Rabbit polyclonal anti-phospho-4E-BP1 (Ser65)	Cell Signaling Technology	Cat# 9451; RRID:AB_330947
Rabbit polyclonal anti-phospho-4E-BP1 (Thr70)	Cell Signaling Technology	Cat# 9455; RRID:AB_330949
Mouse monoclonal anti-β-actin	Sigma-Aldrich	Cat# A2228; RRID:AB_476697
HRP conjugated goat anti-rabbit IgG (H+L)	Abcam	Cat# ab205718; RRID:AB_2819160
HRP conjugated rabbit anti-goat IgG	Dako from Agilent	Cat# P0449; RRID:AB_2617143
HRP conjugated rabbit anti-mouse IgG	Dako from Agilent	Cat# P0260; RRID:AB_2636929
Alexa Fluor 488 goat anti-mouse IgG (H + L)	Thermo Fisher Scientific	Cat# A-11001; RRID:AB_2534069
Alexa Fluor 568 goat anti-rabbit IgG (H + L)	Thermo Fisher Scientific	Cat# A-11011; RRID:AB_143157
Alexa Fluor 594 rabbit anti-sheep IgG (H + L)	Abcam	Cat#ab150184

**Bacterial and virus strains**		

DENV-1 strain Hue-525	Takamatsu et al.^35^	N/A
DENV-2 strain DHF0663	GenBank	GenBank accession no. AB189122
DENV-2 strain NIID 00-43	Ito et al.^36^	GenBank accession no. AB111452

**Chemicals, peptides, and recombinant proteins**		

PNGase F	New England Biolabs	Cat# P0704
Protein A/G UltraLink Resin	Thermo Scientific	Cat# 53133
Cyclohexamide	Wako Chemicals	Cat# 037-20991
Bafilomycin A1 (Baf-A1)	Sigma-Aldrich	Cat# B1793-10UG
MG132	Calbiochem	Cat# 474790
Lactacystin	Sigma-Aldrich	Cat# 100208869
Rapamycin	Funakoshi	Cat# LCL-5000
DAPI (4′,6-Diamidino-2-Phenylindole)	Dojindo	Cat# 340-07971
Methyl Cellulose 4000	Wako Chemicals	Cat# 136-02155

**Critical commercial assays**		

Lipofectamine RNAiMAX	Invitrogen	Cat# 13778075
Lipofectamine 2000	Invitrogen	Cat# 11668027
Lipofectamine LTX and Plus	Invitrogen	Cat# 15338-100
Micro BCA Protein Assay Kit	Thermo Scientific	Cat# 23235
ECL^TM^ Prime Western Blotting Detection Reagents	Cytiva	Cat# RPN2232
Brilliant III Ultra-Fast SYBR Green qRT-PCR Master Mix	Agilent Technologies	Cat# 600886

**Experimental models: Cell lines**		

HuH-7, human hepatoma cells	JCRB	Cat# JCRB0403; RRID:CVCL_0336
HepG2, human hepatoma cells	JCRB	Cat# JCRB1054; RRID:CVCL_0027
A549, human lung epithelial carcinoma cells	JCRB	Cat# JCRB0076; RRID:CVCL_0023
BHK-21, baby hamster kidney cells	JCRB	Cat# JCRB9020; RRID:CVCL_1915
C6/36, *Aedes albopictus* mosquito cells	ATCC	Cat# CRL-1660; RRID:CVCL_Z230
VeroE6, monkey kidney epithelial cells	ATCC	Cat# CCL-81; RRID:CVCL_0059

**Experimental models: Organisms/Strains**		

Mouse: Female AG129 (mice deficient in type I and II interferon receptors)	Marshall BioResources	AG129

**Oligonucleotides**		

RRM2 Stealth siRNA: 5′-UGGAGCGAUUUAGCAAGAAGUUCA-3′	Life Technologies	358093F04
ON-TARGETplus Non-targeting Control Pool	Dharmacon	Cat# D-001810-10-05
ON-TARGETplus Non-targeting siRNA #3	Dharmacon	Cat# D-001810-03-05

**Recombinant DNA**		

pcDNA6/myc-His	Thermo Fisher Scientific	Cat# V22120
pcDNA6/myc-His-RRM2	This study	This study
myc-DDK-tagged human furin	OriGene	Cat# RC204279
pRP[Exp]-Neo-CAG>hMTOR	VectorBuilder	NM_004958.3

**Software and algorithms**		

Graphpad Prism 8	GraphPad Software, Inc.	https://www.graphpad.com; RRID:SCR_002798
BioRender	Scientific Image and Illustration Software	https://www.biorender.com; RRID: SCR_018361
ImageJ	NIH	https://imagej.nih.gov/ij/; RRID: SCR_003070
FIJI/ImageJ	NIH	https://imagej.net/Fiji; RRID: SCR_002285
AlphaFold3	Abramson et al.^37^	https://alphafoldserver.com; RRID:SCR_025885
UCSF ChimeraX	Meng et al.^38^	https://www.cgl.ucsf.edu/chimerax/; RRID:SCR_015872

### Experimental model and study participant details

#### Mice

Five-weeks-old female type I and II interferon receptor knockout mice (AG129) were originally purchased from Marshall BioResources (UK) and maintained in the experimental animal center at Kagoshima University, which is accredited by AAALAC International (Accreditation No.001698). The experimental protocols were approved by the animal experimental committees of Kagoshima University (Permission No. 19–28).

#### Cells and viruses

HuH-7 (JCRB0403), HepG2 (JCRB1054), and A549 (JCRB0076) cell lines were obtained from the Japanese Collection of Research Bioresources (JCRB) Cell Bank in Osaka, Japan, and cultured in Dulbecco’s modified Eagle’s medium (Nissui, Tokyo, Japan) supplemented with 10% fetal bovine serum (FBS; Sigma-Aldrich, St. Louis, MO, USA), 2 mM L-glutamine, 3.151 g/L d-glucose, and 1.5 g/L sodium bicarbonate. BHK-21 (JCRB9020) cells were cultured in minimum essential medium with Earle′s salts (Gibco, Waltham, MA, USA) supplemented with 10% FBS, 2 mM L-glutamine, and 1.5 g/L sodium bicarbonate. All cell lines were cultured at 37°C in a humidified incubator with 5% CO_2_. The DENV strains used in this study included DENV-1 strain Hue-525, isolated during a dengue outbreak in Hue, Vietnam, in 2013^35^; DENV-2 strain DHF0663, isolated from a dengue hemorrhagic fever case in Indonesia; and DENV-2 strain NIID 00–43,^36^ isolated at the National Institute of Infectious Diseases (NIID) in Tokyo, Japan. Viral stocks were prepared in mosquito C6/36 cells (CRL-1660; ATCC, Manassas, VA, USA) and stored at −80°C. Viral titers were determined using a plaque-forming assay in Vero cells (CCL-81; ATCC).

### Method details

#### siRNAs and plasmids

The following siRNAs were used: RRM2 (stealth siRNA: 5′-UGGAGCGAUUUAGCAAGAAGUUCA-3′; Life Technologies, Carlsbad, CA, USA), control (ON-TARGETplus Non-targeting Control Pool siRNA, cat# D-001810-10-05; Dharmacon, Lafayette, IN, USA), and control (ON-TARGETplus non-targeting siRNA #3, cat# D-001810-03-05; Dharmacon). Transfections with siRNAs were carried out using Lipofectamine RNAiMAX reagent (Invitrogen, Carlsbad, CA, USA) in accordance with the manufacturer’s protocol. The plasmid pcDNA6/myc-His-RRM2 was generated by PCR amplification of the RRM2 coding region and subcloning into the pcDNA6/myc-His expression vector (Invitrogen). Plasmids encoding human furin (myc-DDK-tagged human furin; cat# RC204279) were obtained from OriGene (Rockville, MD, USA). Plasmids encoding human mTOR (pRP[Exp]-Neo-CAG>hMTOR[NM_004958.3]) were acquired from VectorBuilder (Chicago, IL, USA). Plasmid DNA transfections were conducted using Lipofectamine LTX reagent (Invitrogen) or Lipofectamine 2000 transfection reagent (Invitrogen) following the manufacturer’s instructions.

#### Antibodies and inhibitors

Antibodies against the following proteins were used in the immunoblots: mouse anti-RRM2 clone 1E1 (cat# WH0006241M1; Sigma-Aldrich), rabbit anti-RRM2 (cat# GTX103193; GeneTex, Irvine, CA, USA), rabbit anti-furin (cat# PA1-062, Thermo Fisher Scientific), rabbit anti-RRM1 (cat# ab137114; Abcam, Cambridge, MA, USA), goat anti-p53R2 (*N*-16, sc-10840, Santa Cruz Biotechnology, CA, USA), mouse anti-flavivirus envelope protein monoclonal antibody (clone 4G2; The Native Antigen Company, Oxford, UK), rabbit anti-dengue virus capsid protein (cat# GTX103343; GeneTex), rabbit anti-dengue virus prM protein (cat# GTX128093; GeneTex), mouse anti-dengue virus prM protein (clone CC5.A9.E10; The Native Antigen Company), mouse anti-dengue virus pr monoclonal antibody (cat# LDG0013YA; LEADGENE), mouse anti-dengue virus NS1 monoclonal antibody (cat# ab41490; Abcam), rabbit anti-mTOR (cat# GTX101557; GeneTex), rabbit anti-LC3I/II (cat# PM036; MBL International, Woburn, MA, USA), rabbit anti-4E-BP1 (cat# 9452; Cell Signaling Technology, Danvers, MA, USA), rabbit anti-phospho-4E-BP1 (Ser65) (cat# 9451; Cell Signaling Technology), rabbit anti-phospho-4E-BP1 (Thr70) (cat# 9455; Cell Signaling Technology), and mouse anti-actin antibody (cat# A2228; Sigma-Aldrich). The appropriate horseradish peroxidase-conjugated secondary antibodies (Dako) were then applied. The protein synthesis inhibitor CHX was obtained from Wako Chemicals (Osaka, Japan). The proteasome inhibitors lactacystin and MG132 were acquired from Sigma-Aldrich and Calbiochem (La Jolla, CA, USA), respectively. The autophagy inhibitor bafilomycin A1 was sourced from Sigma-Aldrich. The mTOR inhibitor rapamycin was obtained from Funakoshi (LCL-5000).

#### siRNA transfection and virus infection

The day before infection, HuH-7, HepG2, and A549 cells were plated in six-well plates at a density of 2 × 10^5^ cells per well and reverse-transfected with either non-targeting control or *RRM2* siRNA using Lipofectamine RNAiMAX (Invitrogen) following the manufacturer’s instructions. The cells were subsequently infected with DENV-1 or DENV-2 at a multiplicity of infection (MOI) of 0.1 for 1 h at 37°C with gentle shaking every 10 min; after this, the virus inoculum was removed, and the cells were washed twice with 1× PBS (−) and incubated with culture medium for 72 hpi. To evaluate the cytotoxicity of *RRM2* siRNA, HuH-7, HepG2, and A549 cells were seeded in 96-well plates at 5 × 10^3^ cells per well and incubated with various concentrations of *RRM2* siRNA at 37°C and 5% CO_2_ for 96 h. Cell viability following *RRM2* siRNA treatment was assessed using the Cell counting kit-8 assay (CCK-8, Dojindo Laboratories, Kumamoto, Japan) according to the manufacturer’s instructions. The optical density at 450 nm (OD_450_) was recorded, and the results are presented as ratios of untreated cells.

#### RT-qPCR

Total RNA was extracted from the cells using an ISOGEN Kit (Nippon Gene, Tokyo, Japan) following the manufacturer’s instructions. Culture supernatants were clarified of detached cells and cell debris using centrifugation at 1,200 × *g* for 10 min at 4°C. Viral RNA was then isolated from 250 μL of the clarified culture supernatants using an ISOGEN-LS Kit (Nippon Gene) according to the manufacturer’s instructions. The concentration and purity of the extracted RNA samples were assessed using a NanoDrop ND-1000 Spectrophotometer (NanoDrop Technologies, Inc., Wilmington, DE, USA). Viral RNA copy numbers in cells and culture supernatants were quantified using Brilliant III Ultra-Fast SYBR® Green qRT-PCR Master Mix (Agilent Technologies, Santa Clara, CA, USA) and primers specific for a highly conserved region of DENV *NS1* among the four DENV serotypes, including the forward primer (5′-GTBCACACHTGGACAGA-3′) and the reverse primer (5′-KGHTATTTGYTTCCACA-3′), as previously reported.^39^ Reactions were conducted in 96-well plates using the CFX Connect™ Real-Time PCR Detection System (Bio-Rad, Hercules, CA, USA). Human GAPDH served as an endogenous control for normalization and was quantified using TaqMan Human GAPDH Control Reagents (Applied Biosystems, Foster City, CA, USA). *Furin* and *RRM2* mRNA expression levels were measured using RT-qPCR using primers and probes from TaqMan Gene Expression Assays (Applied Biosystems) for *furin* (Hs00965485_g1) and *RRM2* (Hs00357247_g1). Target gene expression levels were determined using the 2-ΔΔCt method,^40^ and values were normalized to human *GAPDH* expression as an endogenous control.

#### Virus titration

Infectious virus titers in the culture supernatants of transfected cells were determined using a previously reported fluorescent focus assay.^41^ Briefly, BHK-21 cells were seeded in 48-well plates and incubated for 24 h at 37°C and 5% CO_2_. Cell monolayers were inoculated with 10-fold serial dilutions of virus-containing supernatants and incubated for 1 h at 37°C with gentle shaking every 10 min. The viral inoculum was removed, and the cells were washed with EMEM, then overlaid with EMEM containing 10% FBS and 1% methylcellulose (cat#136–02155, Wako Chemicals, Japan). After 5 days of incubation, the overlay medium was removed, and the cells were washed with cold PBS, followed by fixation with ice-cold absolute methanol for 10 min. Cells were stained to visualize foci of viral infection using a mouse anti-flavivirus envelope protein monoclonal antibody (clone 4G2, The Native Antigen Company) and anti-mouse Alexa Fluor 488 secondary antibody (Thermo Fisher Scientific). Fluorescent foci were counted manually using a BZ-X700 fluorescence microscope (Keyence Co., Osaka, Japan), and infectious virus titers were expressed as fluorescent focus units per mL.

#### Western blotting

Transfected or infected cells were harvested and lysed in radioimmunoprecipitation assay lysis buffer (10 mM Tris, pH 7.4; 150 mM NaCl; 0.5% Nonidet P-40; 5 mM EDTA; 1 mM DTT; and 1% SDS) supplemented with protease and phosphatase inhibitors. Cell lysates were incubated for 1 h on ice and cleared by centrifugation at 13,000 × *g* for 30 min at 4°C. Protein concentrations in total cell lysates were measured using the Micro BCA Protein Assay Kit (Thermo Scientific, Rockford, IL, USA). Total protein (20–60 μg) was loaded with 2× Laemmli sample buffer, separated on a 10%–15% SDS-PAGE gel, transferred to Immobilon-P polyvinylidene difluoride membranes (Merck Millipore, Tullagreen, Cork, Ireland), and subjected to immunoblotting under reducing or non-reducing conditions with the indicated antibodies. Clarified culture supernatants from DENV-1-infected and siRNA-transfected HuH-7 cells were diluted with 4x Laemmli sample buffer and boiled at 95°C for 5 min, then subjected to 12% SDS-PAGE and western blot analysis under reducing conditions. Targeted proteins were detected using ECL Prime western blotting detection reagents (cat#RPN2232, Cytiva, UK) and a Fusion Solo system charge-coupled device imager (Vilber-Loumat, France). The band densities of the specified proteins were determined using ImageJ software (National Institutes of Health, Bethesda, MD, USA) and normalized to the loading control β-actin in the same sample.

#### Determination of DENV E glycosylation status

E protein glycosylation status was determined by treating DENV-infected and non- or siRNA-transfected HuH-7 cell lysates with the peptide N-glycosidase F, PNGase F (Cat# P0704S, New England Biolabs Inc., USA) for 4 h at 37°C according to the manufacturer’s protocol. Samples were then analyzed using 10% SDS-PAGE and western blotting under non-reducing conditions using mouse anti-flavivirus E 4G2 mAb.

#### Immunofluorescence staining and confocal microscopy

For DENV infectivity assays, naive HuH-7 and HepG2 cells were plated on glass coverslips in 24-well plates at a density of 5 × 10^4^ cells per well. After 24 h, the cells were inoculated with viral culture supernatants collected from non-transfected and siRNA-transfected cells and incubated for 1 h at 37°C with gentle shaking every 10 min. The viral inoculum was removed, and the infected cell monolayers were incubated at 37°C in 5% CO_2_. After three days of incubation, the cells were washed twice with ice-cold PBS, fixed, and permeabilized with ice-cold acetone-methanol (1:1) for 10 min. Following two washes with PBS, the cells were stained with a mouse anti-flavivirus envelope protein monoclonal antibody (clone 4G2, The Native Antigen Company) in PBS/0.2% BSA for 1 h, washed three times with PBS/0.2% BSA, incubated with anti-mouse Alexa Fluor 488 secondary antibody (Thermo Fisher Scientific) for 1 h, then washed three times with PBS/0.2% BSA. For verifying the subcellular localization of DENV E protein, HuH-7 cells were grown on glass coverslips in a 24-well plate and transfected with either control siRNA or *RRM2* siRNA (5 nM) before infection with DENV-2 at an MOI of 0.1. At 3 days post-infection, cells were fixed, permeabilized, and co-stained with mouse anti-E 4G2 mAb and markers for various cellular compartments: anti-calnexin (ER), anti-ERGIC-53 (ER-Golgi intermediate compartment), anti-GM130 (*cis*-Golgi), and anti-TGN46 (TGN), then probed with the appropriate Alexa Fluor conjugated secondary antibodies.

For furin and RRM2 confocal microscopy assays, HuH-7 cells were seeded on glass coverslips in a 24-well plate (5 × 10^4^ cells/well) and transfected with either control or *RRM2* siRNA at a concentration of 5 nM before undergoing mock or DENV-2 infection at an MOI of 0.1. After 72 h, the cells were washed twice with cold PBS, fixed with 4% paraformaldehyde for 10 min, and permeabilized with 0.2% Triton X-100 for 10 min. Following incubation with anti-furin and anti-RRM2 antibodies, the cells were washed with PBS/0.2% BSA and incubated with Alexa Fluor 568- and Alexa Fluor 488-conjugated secondary antibodies, respectively. Cell nuclei were stained with 4′,6-diamino-2-phenylindole (DAPI; Dojindo). Confocal microscopy was conducted using a BZ-X700 fluorescence microscope (Keyence Co., Osaka, Japan).

#### Quantitative colocalization analysis

Quantification of colocalization based on acquired confocal microscopy images was performed using BIOP JACoP (Just Another Colocalization Plugin) in Fiji/ImageJ software.^42^ Pearson’s correlation coefficient serves as a quantitative measure of colocalization, relying on pixel intensity correlation between the two fluorescence channels.^43^ The values of Pearson’s coefficient range from −1, indicating no colocalization, to 1, indicating perfect colocalization. A value above 0.5 signifies reliable colocalization between two spectrally separated fluorophores.^43^ Regions of interest (ROI) were manually delineated around individual cells, and Pearson’s correlation coefficient was assessed in each ROI.

#### Immunoprecipitation and cycloheximide chase assays

For immunoprecipitation analysis, HuH-7 cells were plated in 60-mm dishes (5 × 10^5^ cells/dish) and maintained at 37°C in a 5% CO_2_ incubator. The following day, the cells were transfected with 5 μg of pcDNA6/myc-His-RRM2 or an empty vector, pcDNA6/myc-His, using Lipofectamine LTX (Invitrogen). At 72 h post-transfection, the cells were washed twice with ice-cold PBS, then lysed in ice-cold cell lysis buffer (CLB, 50 mM Tris-HCl [pH 8.0], 150 mM NaCl, 12 mM deoxycholate sodium salt, 0.1% SDS, 1% Triton X-100, 0.2 mM sodium vanadate, and 1 mM phenylmethylsulphonyl fluoride). This was followed by centrifugation at 13,000 × *g* for 5 min at 4°C. The supernatants of the cell lysates (300 μL) were incubated with anti-RRM2 antibody (Sigma-Aldrich) overnight on a rotor at 4°C, then mixed with 25 μL of Protein A/G Ultralink resin (cat# 53133, Thermo Scientific) with gentle rotation at 4°C for 2 h. The beads were washed five times with wash buffer (50 mM HEPES [pH 7.9], 250 mM KCl, 0.2% NP40, 0.1% Triton X-100, 0.01% SDS, and 1 mM DTT), resuspended in 2× Laemmli sample buffer, and subjected to SDS-PAGE and western blotting. The expression of RRM2 and furin in the precipitates was characterized by immunoblotting using mouse anti-RRM2 (Sigma-Aldrich) and rabbit anti-furin (Invitrogen) primary antibodies, respectively. The stability of furin was assessed using a cycloheximide chase assay. HuH-7 cells were transfected with the pcDNA6-myc-His-RRM2 expression vector or pcDNA6 (5 μg) using Lipofectamine LTX (Invitrogen). After 3 days, the cells were treated with CHX (100 μg/mL) for the indicated time points before harvesting.

#### Mice experiments

To evaluate the interaction between RRM2 and furin *in vivo*, AG129 mice (*n* = 3 per group) were mock-infected or subcutaneously infected with DENV-2 NIID 00-43 strain (10^4^ focus forming units/mouse). At day 14 post-infection, mice were euthanized, and liver tissues were collected for immunoprecipitation and western blot analysis. Liver tissue lysates were prepared in ice-cold CLB using a power masher II (Nippi, Japan), then incubated with anti-RRM2 antibody (Sigma-Aldrich) overnight on a rotor at 4°C. The mixtures were incubated with Protein A/G Ultralink resin (Thermo Scientific) with gentle rotation at 4°C for 2 h. After washing five times with wash buffer (50 mM HEPES [pH 7.9], 250 mM KCl, 0.2% NP40, 0.1% Triton X-100, 0.01% SDS, and 1 mM DTT), the immunoprecipitates were analyzed by western blotting.

#### Modeling of furin and RRM2 interaction

We utilized AlphaFold3 (https://www.alphafoldserver.com) to analyze and model the protein complex furin-RRM2. AlphaFold3 enables highly accurate structure prediction of protein interactions.^37^ The structures of furin (UniProt: P09958) and RRM2 (UniProt: P31350) were retrieved from the Protein Data Bank UniProt (https://www.uniprot.org/). Visualization of protein interaction structures was performed using UCSF ChimeraX.^38^

#### mTOR inhibition and overexpression assays

For treatment with the mTOR inhibitor rapamycin, HuH-7 cells were seeded at 2.5 × 10^5^ cells per well in a six-well plate and maintained at 37°C and 5% CO_2_. After 24 h, the growth medium was replaced with culture medium supplemented with DMSO or rapamycin (50, 100, or 200 nM). Cells were harvested 12 h after treatment and subjected to protein extraction and western blot analysis. For the combinatorial treatment with *RRM2* siRNA and rapamycin, HuH-7 cells were transfected with either control or *RRM2* siRNA (5 nM), followed by DENV-2 infection at an MOI of 0.1. At 48 hpi, the cells were treated with DMSO or rapamycin for an additional 12 h. In the mTOR overexpression experiment, HuH-7 cells were seeded in six-well plates at a density of 2.5 × 10^5^ cells per well and grown overnight at 37°C with 5% CO_2_ for transfection. The following day, 2.5 μg of mTOR expression plasmid per well was used for transient transfection either alone or co-transfected with 5 nM *RRM2* or control siRNA using Lipofectamine 2000 (Invitrogen) according to the manufacturer’s protocol. At 24 h post-transfection, cells were infected with DENV-2 at an MOI of 0.1, as described above, and then incubated for another 48 h before harvesting for viral RNA quantification and western blot analysis.

### Quantification and statistical analysis

GraphPad Prism version 8.0 (GraphPad Software, San Diego, CA, USA) was used for graph drawing and statistical analysis. Quantitative data are presented as mean ± standard deviation. Student’s *t test* was employed to calculate *p*-values. A *p*-value <0.05 was deemed statistically significant.
