# Supplementary material for: Ribonucleotide reductase subunit M2 mediates the mTOR pathway to recruit furin endoprotease and promote maturation of dengue virus
Source: iScience. 2025 Nov 10;28(12):113998. doi: 10.1016/j.isci.2025.113998 (PMC12670889; doi:10.1016/j.isci.2025.113998)
Supplement: Document S1. Figures S1–S3 [file mmc1.pdf]

iScience, Volume *28*

Supplemental information

Ribonucleotide reductase subunit M2 mediates the mTOR pathway to recruit furin endoprotease and promote maturation of dengue virus

Bouchra Kitab, Michinori Kohara, and Kyoko Tsukiyama-Kohara

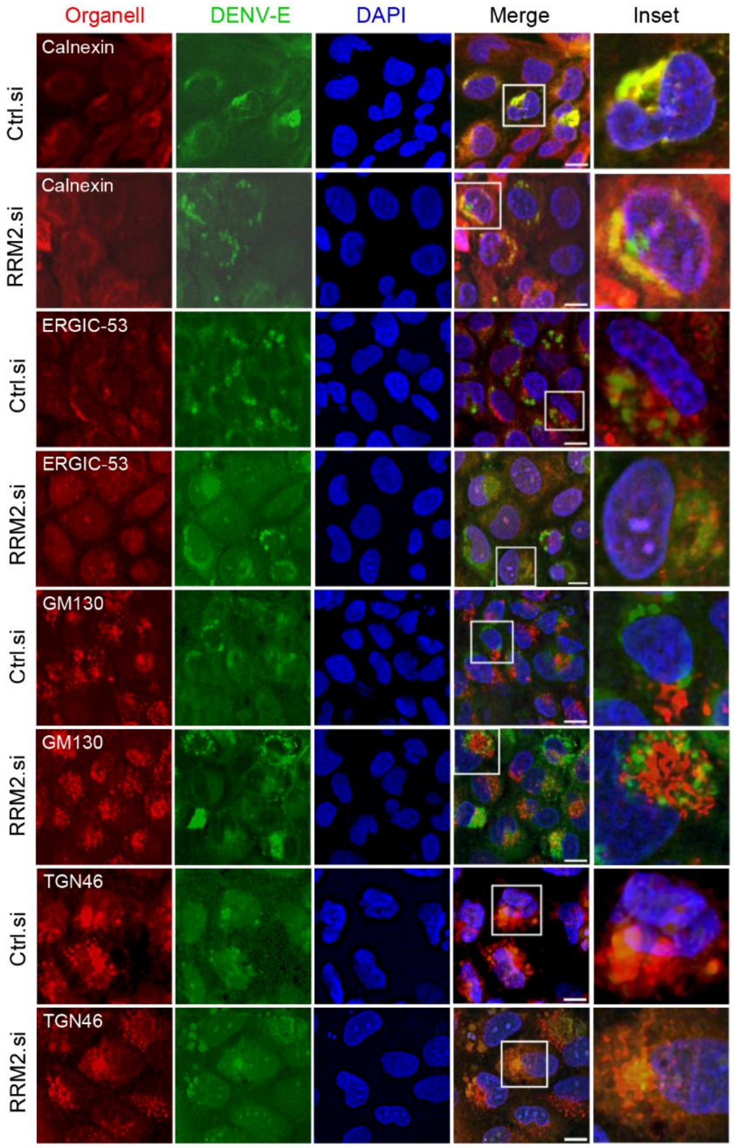

Figure S1. Subcellular localization of the DENV-2 E protein in HuH-7 cells

Cells were infected with DENV-2 at an MOI of 0.1 following transfection with *RRM2* siRNA or control siRNA. On day 3 post-infection, cells were fixed, permeabilized, and co-stained for E protein with mouse anti-E 4G2 mAb (green) and markers for various compartments (red): rabbit anti-calnexin (ER), rabbit anti-ERGIC-53 (ER-Golgi intermediate compartment), rabbit anti-GM130 (*cis*-Golgi), and sheep anti-TGN46 (TGN). Nuclei were visualized using DAPI (blue). Images were visualized using a fluorescence microscope BZ-X700 (Keyence Co., Osaka, Japan) at 200× magnification. Scale bars indicate 100 µm.

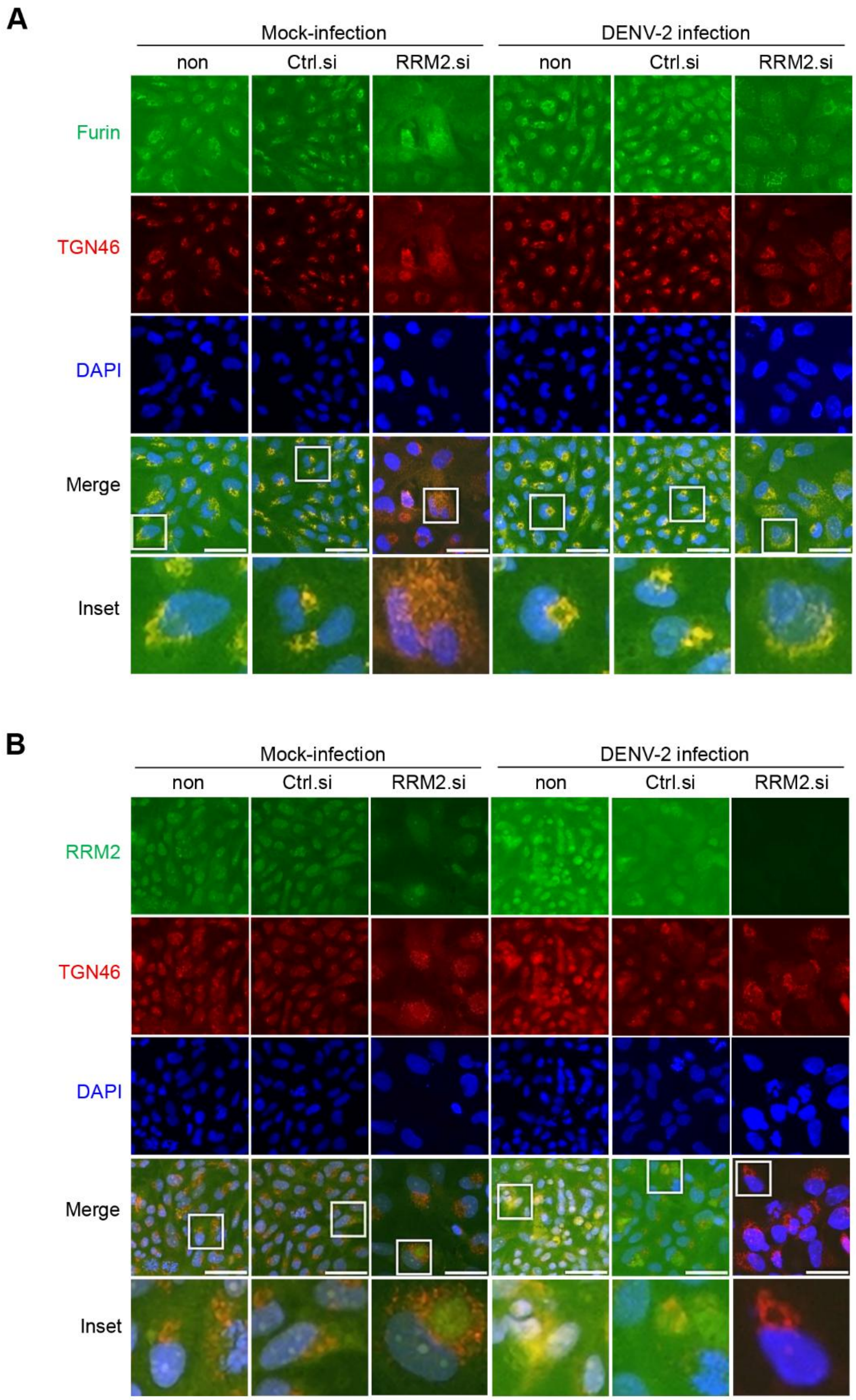

Figure S2. Subcellular localization of RRM2 and furin in HuH-7 cells
Cells were mock-infected or infected with DENV-2 at an MOI of 0.1 following transfection with *RRM2* siRNA or control siRNA. After 72 h, cells were fixed, permeabilized, and co-stained with anti-furin and anti-TGN46 (*Trans*-Golgi network marker) antibodies (**A**), or anti-RRM2 and anti-TGN46 antibodies (**B**). Nuclei were visualized using DAPI (blue). Images were visualized using a fluorescence microscope BZ-X700 (Keyence Co., Osaka, Japan) at 200× magnification. Scale bars indicate 50 μm.

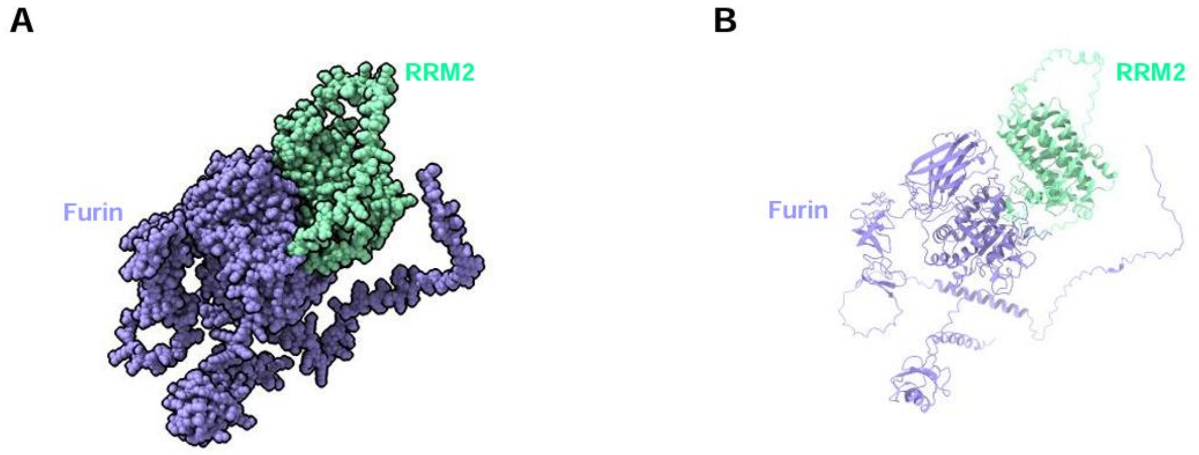

Figure S3. Modeling of RRM2 and furin interaction

(**A**) Surface representation of the RRM2-furin complex. (**B**) Ribbon representation of the RRM2-furin complex. Furin is depicted in purple (aa 1–794, UNIPROT: P09958), and RRM2 is depicted in green (aa 1–389, UNIPROT: P31350). AlphaFold3 was used for modeling.
